# Mixed Etiology COVID-19 Associated Pulmonary Aspergillosis (CAPA)—A Case Report and Brief Review of the Literature

**DOI:** 10.3390/jof7100877

**Published:** 2021-10-18

**Authors:** Dan Alexandru Toc, Carmen Costache, Alexandru Botan, Razvan Marian Mihaila, Ioana Alina Colosi, Sandor Botond Buksa, Roxana Mihaela Chiorescu

**Affiliations:** 1 Department of Microbiology, Iuliu Hatieganu University of Medicine and Pharmacy, 400012 Cluj-Napoca, Romania; carmen_costache@elearn.umfcluj.ro (C.C.); icolosi@umfcluj.ro (I.A.C.); chiorescu.roxana@umfcluj.ro (R.M.C.); 2 Cluj County Emergency Hospital, 400000 Cluj-Napoca, Romania; mihaila.razvan.marian@elearn.umfcluj.ro (R.M.M.); buksaboti@yahoo.com (S.B.B.)

**Keywords:** COVID19, CAPA, IPA, *Aspergillus* section Flavi, *Aspergillus* section Fumigati, Romania

## Abstract

The SARS-CoV-2 pandemic has proved to be a significant risk addition for invasive infections with *Aspergillus.* Even though there are plenty of data about the COVID-19-associated pulmonary aspergillosis (CAPA), especially involving *Aspergillus fumigatus*, recent studies are presenting cases of CAPA involving more than one species of *Aspergillus*. We report the first case of a SARS-CoV-2 patient associating co-infection with, most likely, *Aspergillus* section Fumigati and *Aspergillus* section Flavi from Romania, and we review the existing medical literature in order to shed light upon mixed etiology cases of CAPA. Since mortality remains high in these cases, there is an acute need for more information about the interaction between SARS-CoV-2 and *Aspergillus* spp., and the therapies for CAPA. The emerging number of cases and the high mortality rate must be considered an incentive for future research.

## 1. Introduction

The severe acute respiratory syndrome caused by the coronavirus 2 virus (SARS-CoV-2) is associated with severe lung injury and complex inflammatory responses [1]. Most therapies for this disease use corticosteroids and immunomodulatory agents, causing a decrease of the immune response and, therefore, the possibility of other pathogens to colonize and produce a wide variety of infections, thus influencing the outcome of the disease [2].

There are numerous cases of co-infections with microbial and viral pathogens, but fungal co-infections are rarely reported. There is an increasing number of reported cases of invasive infections with *Aspergillus* spp. in SARS-CoV-2 patients. Most cases of COVID-19 associated pulmonary aspergillosis (CAPA) end up not being reported due to the diagnostic difficulties, as well as the lack of clinical awareness. The high mortality rate of the disease generated major interest in the association between the pathophysiological processes produced by fungal–virus interaction [3].

*Aspergillus fumigatus*, one of the most studied species of all *Aspergillus*, is known to be the most common pathogen causing invasive aspergillosis. *Aspergillus flavus*, on the other hand, was proved to be more virulent than *Aspergillus fumigatus*, even though they cause similar clinical syndromes. The existing data suggest that there might be a geographic difference in the distribution of these pathogens with most cases of *Aspergillus fumigatus* infections being reported in Western Europe, while *Aspergillus flavus* was more common in Asia, the Middle East and Africa. However, in order to shed light on this characteristics, more epidemiological studies are needed [3,4].

To the best of our knowledge, this paper presents the first case of CAPA with mixed fungal etiology from Romania and one of the few cases reported worldwide. This aims to contribute to the literature regarding the diagnosis, management of these cases, and the geographical distribution of the disease. CAPA remains a poorly defined entity and, with the new variants of SARS-CoV-2 emerging worldwide, we are facing a race against the clock to improve the management of this disease.

## 2. Case Presentation

A 53-year-old woman presented at the emergency room with a 4-day history of fever, headache, fatigue, dizziness and productive cough. She had a medical history of type 2 diabetes mellitus, asthma, chronic kidney disease, grade I obesity (BMI = 32.87 kg/m^2^), Hashimoto thyroiditis, ischemic heart disease, severe pulmonary hypertension, and mitral valve stenosis post prosthetic mitral valve implantation. Her treatment consisted of: spironolactonum/furosemidum, nebivololum, insulinum glargine, levothyroxinum, beclometasonum/formoterolum, acenocumarolum. Her husband was COVID-19 positive and she claimed a close household contact with him.

Rapid antigen testing for SARS-CoV-2 was positive at admission. To confirm the SARS-CoV-2 infection, a nasopharyngeal swab sample was collected in order to perform the molecular testing using RT-PCR.

At admission, physical examination showed a hemodynamically and respiratory stable patient with the following parameters: blood pressure = 139/79 mmHg, heart rate = 69 beats per minute, SaO2 = 88% on room air, body temperature = 36.4 °C.

Thoracic radiography revealed sternal threads after valve replacement surgery, no pleural collections, no pulmonary condensation, grade II venous stasis, cardiomegaly and metal prosthesis in mitral position.

Abdominal, thyroid and cardiac ultrasounds were performed on the 3rd day of hospitalization. The abdominal ultrasound showed dilatation of the inferior vena cava and suprahepatic veins, the gallbladder with a hyperechoic image. The cardiac ultrasound revealed a normofunctioning prosthetic valve, left atrium dilatation (50 mm), mild interventricular septal dyskinesia, severe secondary pulmonary hypertension, normal ejection fraction and monophasic mitral flow. The thyroid ultrasound detected a 1 cm/1.3 cm hypoechoic thyroid nodule for which we recommended further endocrinological evaluation.

A native thoraco-pulmonary CT exam revealed the image of a typical viral pneumonia with a degree of lung damage of approximately 10%. This was interpreted as a mild form of SARS-CoV-2 pneumonia. Because the RT-PCR from the nasopharyngeal swab was positive for SARS-CoV-2, we established the main diagnosis of bilateral COVID-19 pneumonia and acute respiratory failure. We initiated antiviral treatment with Remdesivir (Veklury^®^) 200 mg on the first day, and 100 mg over the next four days. The summary of medication used in the hospital is presented in Table 1.

Although initial ECG showed sinusal rhythm with 60 beats/min without other modifications, 24 h after the admission ECG exploration revealed atrial fibrillation with 208 beats/min for which we initiated the treatment following the guidelines with 1200 mg/24 h of amiodarone. Unfortunately, we could not convert this to normal sinus rhythm. We continued the administration of amiodarone during hospitalization.

The evolution of the laboratory tests and the general presentation of the patient management is presented in Table 2.

**Figure 1:**
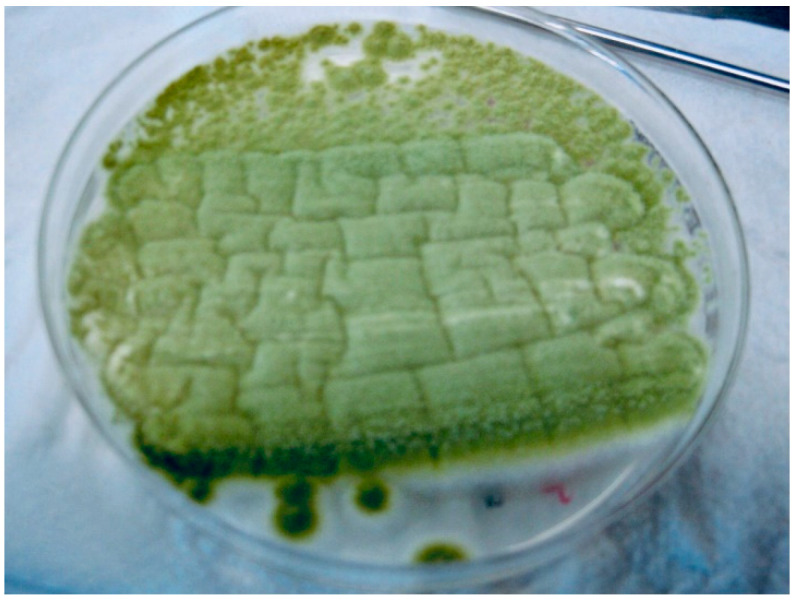
Culture of *Aspergillus flavus* on Sabouraud chloramphenicol gentamicin agar (Bio-Rad, Marnes-la-Coquette, France).

**Figure 2:**
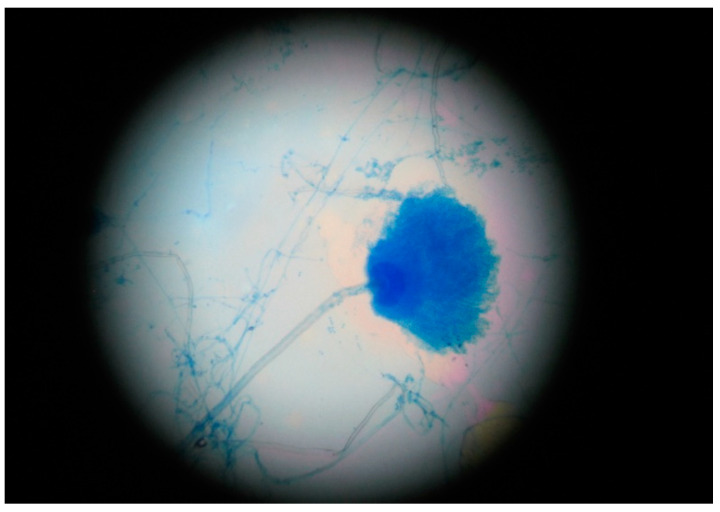
*Aspergillus flavus*, wet mount with methylene blue (cellophane tape mount), 400×, Optic microscope (Karl Zeiss, Jena, Germany).

On the 5th day of hospitalization, microbiologic results from the sputum sample collected on the 1st day came back negative for bacteria. However, macroscopic examination of the fungal culture on Sabouraud agar shows several wide, flat, floccose colonies with a yellow-green color. In order to ensure the isolation and identification of the fungi, aspergilli were subculture from the primary culture on Sabouraud chloramphenicol gentamicin agar (Bio-Rad, Marnes-la-Coquette, France) (Figure 1). Microscopic examination, using the lactophenol cotton blue method, revealed vesiculated conidiophores with large, round and smooth conidia, roughened stalks, phialides arising directly from the entire surface of the vesicle in some heads, and produced on metulae in others (Figure 2). These findings concluded in the identification of *Aspergillus* spp. most likely *Aspergillus* section Flavi. The antifungal susceptibility testing, using the concentration gradient strip Etest method, revealed that this strain was susceptible to voriconazole. We collected another sputum sample and initiated intravenous specific antifungal treatment with voriconazole: 2 × 400 mg at first day, 2 × 200 mg the next days. After 5 days, the antiviral therapy with Remdesivir was stopped.

**Figure 3:**
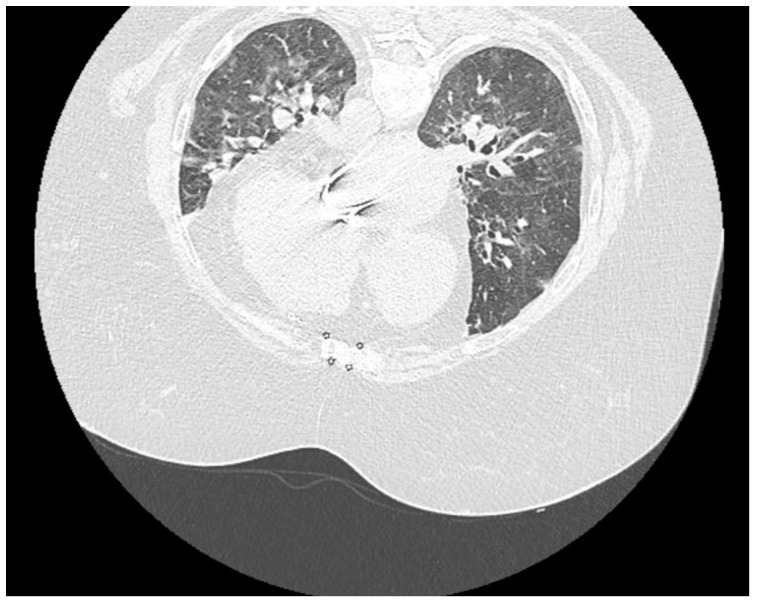
Native thoraco-pulmonary CT of the patient, revealing a viral SARS-CoV-2 pneumonia with a degree of damage around 50–60% and with unsystematized central and peripheral pulmonary infiltrates and ground-glass opacities.

On the 8th day of hospitalization, we repeated the native thoraco-pulmonary CT examination (Figure 3), which showed unsystematized central and peripheral pulmonary infiltrates and ground-glass opacities with a degree of damage of around 50–60%. These findings were suggestive of a severe form of SARS-CoV-2 pneumonia. There were also several mediastinal lymph node images, enlarged heart, the valvuloplasty in the mitral position and trachea-bronchial malformations. Pulmonary condensation foci were described in upper right lobe, upper left lobe and lower left lobe. Also, at the level of the upper abdomen included in the examination, a 15/23 mm nodular lesion was highlighted in the left adrenal gland.

**Figure 4:**
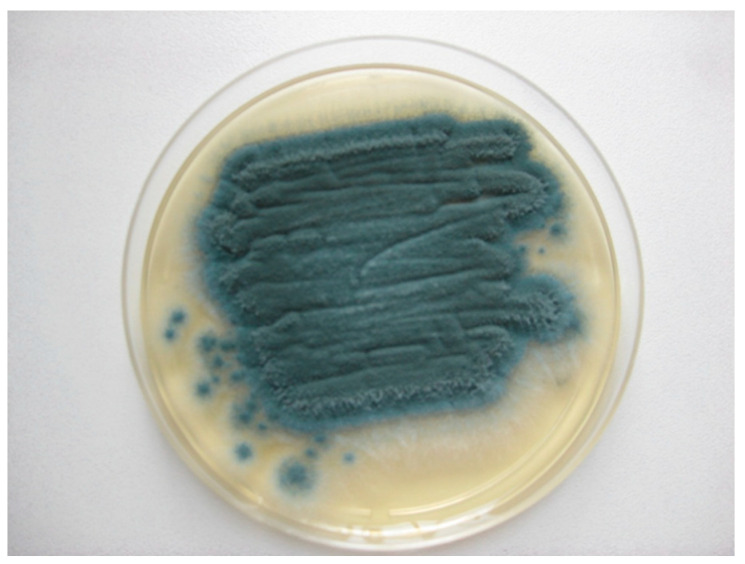
Culture of *Aspergillus fumigatus* on Sabouraud chloramphenicol gentamicin agar (Bio-Rad, Marnes-la-Coquette, France).

**Figure 5:**
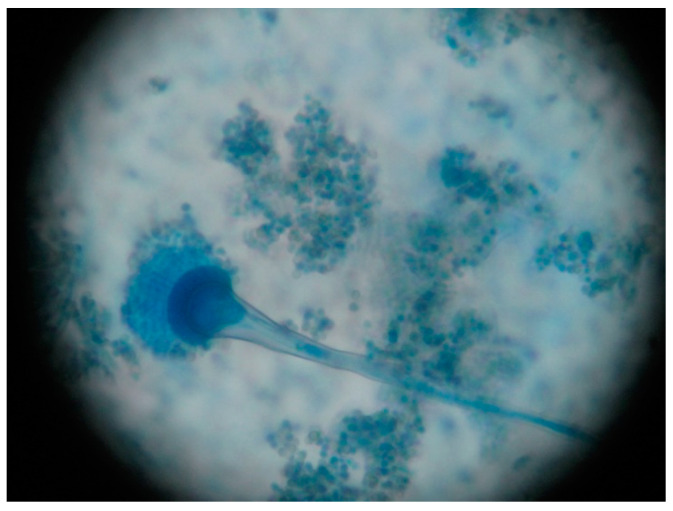
*Aspergillus fumigatus*, wet mount with methylene blue (cellophane tape mount), 400×, Optic microscope (Karl Zeiss, Jena, Germany).

On the 10th day of hospitalization, the microbiological report from the sputum sample collected on the 5th day came back negative for bacteria and positive for fungi. Macroscopic examination of the fungal culture on Sabouraud agar showed a flat, powdery colony with a blue-green color. In order to ensure the isolation and identification of the fungi, aspergilli were subcultured from the primary culture on Sabouraud chloramphenicol gentamicin agar (Bio-Rad, Marnes-la-Coquette, France) (Figure 4). Microscopic examination, using lactophenol cotton blue method, revealed vesiculated conidiophores with small, round and slightly roughened conidia, short stalks, pear-shaped vesicles, crowded phialides arising from the upper two-thirds of the vesicles only with no metulae (Figure 5). These findings concluded the diagnostic of *Aspergillus* spp., most likely *Aspergillus* section Fumigati. The antifungal susceptibility testing, using the concentration gradient strip Etest method, revealed that this strain was also susceptible to voriconazole. Before we again started the intravenous treatment with voriconazole, we collected blood to determine the level of galactomannan, which is the serological test with the best sensitivity used for demonstrating the presence of *Aspergillus* antigen. Galactomannan’s index was 0.65 which according to the product specifications it was reactive for *Aspergillus* antigen [5,6,7].

On the 13th day of hospitalization, we have repeated the RT-PCR test from a nasopharyngeal swab. The result was negative for SARS-CoV-2 and the patient was discharged the next day with the following recommendations: continuing voriconazole therapy for 5 weeks more, maintaining physical distance, wearing the mask at home and at work for another 10 days after the end of the isolation period, avoiding close contact with other people in the house and, if possible, isolation in a separate room with separate bathroom, washing hands with soap and water for at least 20 s and frequent use of disinfectants.

## 3. Discussion

We presented the first reported case of COVID-19 Associated Pulmonary Aspergillosis (CAPA) with mixed (*Aspergillus* section Flavi and *Aspergillus* section Fumigati) from Romania and one of the few cases reported worldwide with a positive outcome.

At the moment, diagnosing CAPA poses a great challenge. There are several guidelines that can help in the diagnostic process but, even so, they are difficult to apply in some cases, thus delaying the commencement of the antifungal treatment. The proven cases of CAPA involve histopathologic evaluations of tissue samples either post mortem or from the biopsy material taken from a bronchoscopy. Although bronchial brush or aspirate are preferable, sputum is accepted as sample for diagnosis of lower respiratory tract infections [8].

Mohamed A et al. present what we consider the optimal approach in this case in order to begin the antifungal therapy on time. In order to diagnose CAPA they suggest combining two or more of the following mycological criteria: serum galactomannan (GM) detection, isolation of *Aspergillus* spp., serum 1–3 β-D-glucan (BDG) test and detection of *Aspergillus* spp. DNA by real time PCR [9]. Using this algorithm, we were able to diagnose our case of mixed etiology CAPA and proceed with the antifungal treatment on time, thus saving the patient’s life. One of our main concerns regarding this case was the risk of fungal endocarditis due to the patient’s history of cardiac disease. Fortunately, the patient responded to the treatment and no cardiac involvement was noted.

Since the beginning of the pandemic, invasive pulmonary aspergillosis (IPA) was a foreseeable complication of COVID-19. What we learned from the influenza and the SARS-CoV-1 outbreaks is that co-infection with a fungal pathogen represents a great threat to human health. The reported mortality in these situations is up to 67% in some studies [10].

There are many risk factors that may be involved and there is a great impetus worldwide to understand the pathogenicity of CAPA. Some of the most relevant existing data described the implications of the IL-1, IL-6 and IL-10 in the pathogenicity and evolution of CAPA. Caglar et al. demonstrated that *Aspergillus* infection is associated with an elevated level of IL-10 but in IPA patients, the high level of IL-10 seems to lead to a stimulation of Th-2 cells followed by a depression of Th-1 cells which ultimately can cause invasive aspergillosis [11]. Regarding the IL-6, a known cytokine with an important role in anti-*Aspergillus* immunity, existing data suggests that in CAPA patients there is an elevated level of IL-6 but a reduced responsiveness of T cells [2]. The IL-1 pathway is described as being upregulated and, therefore, influencing the hyperinflammation present in cases of COVID-19. Also, early involvement of IL-1 promotes a favorable medium for *Aspergillus* to colonize the lungs, thus the risk for CAPA. There seems to be no doubt that further studies can lead us to exciting new treatment options, finding the best way to use the immunomodulators in the treatment of CAPA [12].

Regarding the limitations of our paper, the first we would underline is the lack of molecular diagnosis of the *Aspergillus* species involved. Although some of the most widely used markers for molecular diagnosis are beta-tubulin, and elongation factor, since the molecular diagnosis is not always available in developing countries, the fungal culture and microscopic description remain valid methods to be used, in order to identify, although not definitively, different fungi [4,13,14,15]. Another relevant limitation is represented by the pathological product from which the *Aspergillus* species in our paper were identified. Regarding the sputum sample, trying to differentiate a species that only colonizes the respiratory tract from one that is involved in the pathology remains a challenge. We were able to overcome this issue with the GM index which suggested that we were facing an infection rather than a colonization.

We believe that in order to better understand the implications of such a unique synergy between two human pathogens, it is of utmost importance to have a global perspective regarding the diagnosis and management of these cases and thus we performed a review of the medical literature of the mixed etiology CAPA cases reported. We searched on PubMed and Cochrane library electronic databases, up to 15 August 2021. We considered the following terms included in the studies’ title or abstract: “aspergillus”,” CAPA” combined with the Boolean operator” AND” along with” human” and” COVID-19”. We excluded studies written in languages other than English and French. The results are summarized in Table 3.

We found only eight cases of mixed etiology CAPA across six articles, including the one presented in this paper. *Aspergillus fumigatus* was involved in all cases of mixed etiology CAPA we reviewed. The association of *Aspergillus flavus* and *Aspergillus fumigatus* was present in three cases, being the most frequent association reported, followed by *Aspergillus niger* and *Aspergillus fumigatus* present in two cases. There is also one reported case of CAPA with *Aspergillus fumigatus*, *Aspergillus terreus* and *Aspergillus awamori,* suggesting that multiple associations of *Aspergillus* are possible in cases of CAPA. Mixed etiology cases of CAPA that involved the association of *Aspergillus fumigatus* and *Aspergillus flavus* had an unfavorable outcome, the present case being the only one where the patient recovered. Other associations like *Aspergillus fumigatus* and *Aspergillus terreus* or *Aspergillus niger* were followed by a more favorable outcome. Regarding the treatment, isavuconazole and voriconazole were the most frequently used drugs, with variable outcomes. Antifungal treatment can contribute to significant drug interactions and physicians should always balance the risks and benefits when choosing a particular agent.

Although the existing data are scarce, we believe that rapid diagnosis of CAPA and prompt initiation of antifungal treatment can improve the outcome. However, we hypothesize that *Aspergillus* species involved in cases of mixed etiology CAPA can also influence the outcome and further studies are required in order to shed light upon this new threat.

## Figures and Tables

**Table 1:** Medication used for the treatment.

International Nonproprietary Name (INN)	Dose	Details
REMDESIVIRUM	100 mg	Day 1–5
VORICONAZOLUM	200 mg	Day 5–14
ACENOCUMAROLUM	4 mg	Day 10–14
INSULINUM GLARGINE	14 UI	Day 1–14, adjusted according to glicemic profile
SPIRONOLACTONUM/FUROSEMIDUM	50/20 mg	Day 1–14
NEBIVOLOLUM	5 mg	Day 1–14
LEVOTHYROXINUM	100 µg	Day 1–14
BECLOMETASONUM/FORMOTEROLUM	100/6 µg	Day 1–14
DEXAMETHASONUM	8 mg	Day 1–14
FAMOTIDINUM	20 mg	Day 1–14
LACTOBACILLUS PARACASEI CNCM I-1572	3 g	Day 1–14
ENOXAPARINUM	60 mg/0.6 mL	Day 1–14
DIGOXINUM	0.5 mg	Day 1–14
MONTELUKASTUM	10 mg	Day 1–14
AMBROXOLUM	30 mg	Day 1–14
CEFTRIAXONUM	2 g	Day 1–10
DOXYCYCLINUM	100 mg	Day 1–10

**Table 2:** Evolution of the laboratory tests and general presentation of the patient management.

		Day 1	Day 2	Day 3	Day 4	Day 5	Day 8	Day 9	Day 10	Day 11	Day 12
Clinical signs and Imaging		Fever, headache, fatigue, dizziness Hemodynamic and respiratory stabile CT: ≈10%	Systemic Inflammatory Response Syndrome (SIRS)	Complementary paraclinical examinations	Nitrogen retention syndrome and SIRS decrease		CT ≈50-60% Severe form of SARS-CoV-2 pneumonia				
Laboratory	Hematological	WBC: 6.720 cells/mm^3^ Neutrophils: 78% Lymphocytes: 13.8% RBC: 5,431,000 cells/mm^3^ Fibrinogen: 653.6 mg/dL	Neutrophils: 89.9% Lymphocytes: 6.2%		Neutrophils: 89% Lymphocytes: 6%	Hb: 15.4 g/dL pH: 7.54 pCO_2_: 55.4 mmHg SaO_2_: 91.4%	Hb: 15.4 g/dL pH: 7.54 pCO_2_: 55.4 mmHg SaO_2_: 91.4%			WBC: 12,730/mm^3^ Neutrophils: 87.3% Lymphocytes: 5.2%	
	Metabolism	Glucose: 116 mg/dL		Glucose: 235 mg/dL		Glucose: 319 mg/dL Lactate: 15 mg/dL	Glucose: 319 mg/dL Lactate: 15 mg/dL		Glucose: 338 mg/dL	Ferritin: 1109 ng/mL Glucose: 327 mg/dL	
	Liver function	ASAT: 46 IU/L GGT: 214 IU/L LDH: 458 IU/L Direct bilirubin: 0.33 mg/dL C-reactive protein: 19.29 mg/dL	C-reactive protein: 19.29 mg/dL INR: 5.26		C-reactive protein: 9.77 mg/dL	C-reactive protein: 1 mg/dL	C-reactive protein: 1 mg/dL	ASAT: 25 IU/L, ALAT: 31 IU/L C-reactive protein: 0.65 mg/dL		C-reactive protein: 0.65 mg/dL	
	Kidney function	BUN: 58 mg/dL Creatinine: 1.86 mg/dL Uric acid: 13 mg/dL	BUN: 70 mg/dL Creatinine: 1.59 mg/dL eGFR: 36.41 mL/min/1.73m^2^		Creatinine: 1.12 mg/dL	BUN: 63 mg/dL Creatinine: 1.07 mg/dL eGFR: 58.78 mL/min/1.73m^2^	BUN: 63 mg/dL Creatinine: 1.07 mg/dL eGFR: 58.78 mL/min/1.73m^2^	BUN: 60 mg/dL Creatinine: 1.03 mg/dL eGFR: 81.55 mL/min/1.73 m^2^		BUN: 60 mg/dL Creatinine: 1.03 mg/dL eGFR: 91.2 mL/min/1.73 m^2^	
Microbiology		RT-PCR positive for SARS-CoV-2 Sputum sample collection				(+) *Aspergillus section Flavi* (−) Bacteriological Sputum sample collection			*Aspergillus* section *Fumigati* detected Galactomannan’s index: 0.65 Aspergillus antigen present		Galactomannan’s index: 0.65 Aspergillus antigen present

Abbreviations: ALAT, alanine aminotransferase; ASAT, aspartate aminotransferase; BUN, blood urea nitrogen; CT, computed tomography; eGFR, estimated glomerular filtration rate; INR, international normalized ratio; LDH, lactate dehydrogenase; RBC, red blood cells; RT-PCR, reverse transcription polymerase chain reaction; SARS-CoV-2, severe acute respiratory syndrome coronavirus-2; WBC, white blood cells; (+), positive; (−), negative; Hb, hemoglobin; pCO_2_, partial pressure of carbon dioxide; pH, potential of hydrogen; SaO_2_, oxygen saturation.

**Table 3:** Review of the existing case reports of COVID-19 associated pulmonary aspergillosis (CAPA) involving a mixed fungal etiology.

No.	Study	Year	Country	Patient (Age/Gender)	Species	Sample	GMI	BDG (pg/mL)	Anti-Fungal Treatment	Outcome	Diagnosis
1	Machado M et al. [3]	2020	Spain	63/M	*A. fumigatus* + *A. awamori* + *A. terreus*	Bronchial aspirate	0.56	5.4	Isavuconazole (starting date not specified)	Death	Morphologic identification Galactomannan (GM) (1,3)-β-d-glucan (BDG) PCR
2	Nasir N et al. [16]	2020	Pakistan	57/M	*A. flavus* + *A. fumigatus*	Sputa or tracheal aspirate	0.272	<7	Amphotericin B (started on day 5)	Death	Morphologic identification Galactomannan (GM) (1,3)-β-d-glucan (BDG)
3	Abolghasemi S et al. [17]	2021	Iran	66/F	*A. Terreus* + *A. fumigatus* (low colony count)	Mini-bronchoalveolarlavage	4.15	Not done	Voriconazole Caspofungin (started on day 21)	Death	Morphologic identification Galactomannan (GM) PCR
4	Marr K.A et al. [18]	2021	Spain	70/M	*A. fumigatus* + *A. niger*	Tracheal aspirate/sputum	Not done	Not done	Voriconazole, posaconazole, or liposomal amphotericin B (starting date not specified)	Survived	Morphologic identification
				76/M	*A. fumigatus* + *A. niger*	CT scan	Not done	Negative		Survived	Morphologic identification
5	Paramythiotou E et al. [19]	2021	Greece	82/F	*A. fumigatus* + *A. flavus*	Bronchial secretions	9.9	913.7	Isavuconazole (started on day 11)	Death	Morphologic identification Galactomannan (GM) (1,3)-β-d-glucan (BDG) PCR
				66/M	*A. fumigatus* + *A. terreus*		11.4	906.7	Isavuconazole (started on day 6)	Survived	
6	This study	2021	Romania	53/F	*Aspergillus* section *Fumigati* + *Aspergillus* section *Flavi*	Sputum	0.65	Not done	Voriconazole (started on day 5)	Survived	Morphologic identification Galactomannan (GM)

Abbreviations: BDG, β-d glucan assay; CT, computed tomography; GMI, Galactomannan Index.

## Data Availability

Not applicable.
